# Smoking behavior among Asian Americans during the initial phase of the COVID-19 pandemic: The influence of pandemic stressors and depression

**DOI:** 10.18332/tid/176923

**Published:** 2024-01-25

**Authors:** Paula Lozano, Aven Peters, Alia Southworth, Yicklun Mo, Helen Lam, Fornessa T. Randal, Karen E. Kim

**Affiliations:** 1 Department of Biomedical Sciences, Center for Asian Health Equity - University of Chicago Medicine, Chicago, United States; 2 Asian Health Coalition, Chicago, United States

**Keywords:** Asian Americans, current smoking, COVID-19, pandemic-related stressors, discrimination, depression

## Abstract

**INTRODUCTION:**

Heightened levels of distress among Asian Americans during the initial phases of the pandemic may be associated with current smoking behavior. In this study, we examine differences in current smoking among Asian Americans from two different ethnic backgrounds before and during the COVID-19 pandemic.

**METHODS:**

We analyzed cross-sectional survey data (n=202) from Chinese and South Asian adults in Chicago, collected between February and May 2020. We conducted logistic regression models to estimate the relationship between exposure to the COVID-19 pandemic and current smoking. We tested whether the association varied by Asian American ethnic group, unemployment, racial discrimination, and depression symptoms.

**RESULTS:**

We found that current smoking increased from 28% to 48% among Asian Americans (i.e. Chinese and South Asians) during the pandemic. We found a statistically significant interaction between the COVID-19 period indicator variable and current smoking by Asian American ethnic groups (p=0.014), such that current smoking was lower for Chinese compared to South Asians before COVID-19, but was comparable for both groups during the pandemic. We also found a statistically significant interaction between the period indicator variable and current smoking by racial discrimination (p=0.047) and depression symptoms (p=0.02). Results from these interactions suggest that Asian Americans who experienced racial discrimination and depression during the pandemic may be more likely to be current smokers compared to their pre-pandemic counterparts.

**CONCLUSIONS:**

The findings of the study highlight the need for culturally tailored smoking cessation interventions for Asian American communities that address pandemic-related stressors such as discrimination that may trigger cigarette use.

## INTRODUCTION

Multiple studies have found increased smoking rates during the COVID-19 pandemic compared to before the pandemic^1-4^. It is possible that COVID-19-related stressors, such as feelings of isolation, loneliness, and financial problems, are associated with smoking behaviour^1,4^. Among Asian Americans, these stressors may be compounded by heightened levels of anxiety and depression caused by perceived racial/ethnic discrimination experienced by these communities during the pandemic^5-8^. Racial discrimination towards Asian Americans was exacerbated by the COVID-19 pandemic^6,7^. The Atlanta spa shooting in which eight people were killed, six of whom were women of Asian descent, depicts the increased verbal and physical violence against this population and the anti-Asian sentiment triggered by COVID-19^9^. Thus, heightened levels of distress among Asian Americans during the initial phases of the pandemic may be associated with current smoking behaviour^8^. In this study, we examine an increase in the differences in current smoking before and during the pandemic using disaggregated data of Asian Americans from diverse ethnic backgrounds.

Although several studies have found an increase in post-pandemic smoking behaviour^1-4^, those that have examined this association by race/ethnicity (i.e. Blacks, Whites, Hispanics, and American Indians) have found mixed results^10,11^. One study found a decrease in smoking among Asian Americans compared to other racial/ethnic groups^10^. However, the use of aggregated Asian data in this study may have obscured important distinctions in cultural values, socioeconomic status, and immigration^12^ that may directly influence smoking behaviour^13^. For instance, in most Asian American cultures, smoking is considered socially unacceptable among women^14^. Thus, the prevalence of current smoking among Asian American women with low levels of acculturation is generally lower^14-17^. Previous studies suggest that cigarette smoking rates vary significantly across Asian American ethnic groups^13,18-21^. A recent study using data from the Nation Health Interview Survey (2006–2018), a telephone-based national study, suggests that current smoking was highest among Filipinos (12.4%) and lowest among Asian Indians (5.1%) and Chinese (5.9%)^20^. Smoking behavior may also differ by contextual factors such as geographical location. A population-based survey among a Chinese enclave (aged 40–69 years) in Chicago found smoking prevalence as high as 34% for adult males^21^. That study found that smoking behavior was driven by risk perceptions and attitudes towards cigarette use^21^. Smoking rates also vary significantly by socioeconomic status, proficiency in the English language, and contextual factors such as discrimination^11,21^. For instance, a study found that current and former smoking was higher among Asian Americans who had experienced COVID-19-related discrimination^11^. Thus, to inform and culturally develop tobacco intervention programs that seek to reduce smoking behavior in this population, it is important to understand the smoking patterns among Asian American populations from diverse ethnic backgrounds and the factors that may influence smoking. In particular, considering the heightened levels of distress among Asian Americans during the initial phases of the pandemic, it is important to understand the role of COVID-19 in smoking behavior among Asian American communities.

In this study, we aim to examine differences in current smoking among Asian Americans from two different ethnic and cultural backgrounds (i.e. South Asians and Chinese) before and during the COVID-19 pandemic, in Chicago. In particular, South Asians and Chinese are important groups to study as they make up the largest population of Asian Americans in the Chicago area^22^. A secondary aim of this study is to evaluate the moderating effect of pandemic-related stressors (i.e. unemployment, discrimination) and depression on the association between COVID-19 exposure (i.e. before vs during the pandemic) and current smoking among Asian Americans. It is possible that unemployed Asian Americans during the pandemic may have turned to smoking to cope with distress due to difficulties finding work and/or economic loss^23^. Moreover, individuals experiencing depression during the pandemic may be at greater risk of smoking. A study examining smoking behavior following the COVID-19 lockdown found that individuals with anxiety and depression were more likely to report increased tobacco use^23^.

## METHODS

### Study design and sample

Data were drawn from the Chicago Asian Health Survey (CAHS), a cross-sectional survey of Asian Americans in Chicago^8^. This survey was adapted from the Healthy Chicago Survey, an annual survey led by the Chicago Department of Public Health to evaluate the health of the population in Chicago^24^. The main goal of this survey was to collect disaggregated health information among eight Asian American (i.e. Cambodian, Chinese, Filipino, Korean, Laotian, Mongolian, South Asian, Vietnamese) communities in Chicago. However, of the eight communities surveyed, only the Chinese and South Asian communities (sampled from February to May 2020) had data before and during the COVID-19 lockdown. Therefore, in this study, we examine the impact of the COVID-19 lockdown on changes in smoking among South Asians and Chinese.

Using convenience sampling, we partnered with four community-based organizations (CBOs) that serve the Chinese and South Asian communities (two CBO partners per community). Each CBO used an individualized, community-based approach to reach its community (e.g. community events and social media posts). Before distributing the survey to the study population, partner Asian American-serving CBOs staff revised the survey for linguistic and cultural appropriateness. To accommodate participants’ language needs, the survey was translated into different languages per partner CBO’s request. More information on the recruitment and data collection process is available elsewhere^8^. The IRB at the University of Chicago determined that our study did not need ethical approval and an official waiver of ethical approval was granted from the IRB at this institution.

### Variables

*Smoking behavior*

Respondents were first asked whether they had smoked at least 100 cigarettes in their entire lives. Response options for this question included ‘Yes’, ‘No’, and ‘I have never smoked cigarettes’. Those who responded ‘Yes’ were then asked how frequently they currently smoked: ‘every day’, ‘some days’, or ‘not at all’. If respondents indicated that they smoked every day or some days, they were classified as current smokers^25^.

*Sociodemographic variables*

Sociodemographic factors included Asian American ethnic group (Chinese, South Asian), sex (male, female), age (18–29, 30–65, >65 years), education level (less than high school, high school or GED, some college, college or more), employment status (employed, not employed) and country of birth (US born, foreign born). These sociodemographic variables were included as confounders in the multivariable logistic regression models, as they have been found to predict smoking behavior among Asian Americans^14-17^.

*Depression symptoms*

Depression symptoms were assessed using the Patient Health Questionnaire eight-item depression scale (PHQ-8)^26-28^. The items in the PHQ-8 ask about the presence of symptoms in the last two weeks^27^. Items are scored from 0 to 3 with response options ‘not at all’ (score 0), ‘several days’ (score 1), ‘more than half the days’ (score 2), and ‘nearly every day’ (score 3). The scores for each item are summed to produce a total score between 0 and 24 points. The binary classification of depression symptoms was defined by a score of ≥10. A previous study suggests that the PHQ-8 score of ≥10 has a sensitivity of 88% and specificity of 88% for major depression^27^.

*Racial discrimination*

To assess racial discrimination, we used the Experiences of Discrimination (EOD), widely used to measure racial discrimination^29^. Participants were asked if they had ever experienced discrimination, were prevented from doing something, hassled or made to feel inferior in any of the following nine areas because of their race, ethnicity, or color: 1) at school; 2) getting hired or getting a job; 3) at work; 4) getting housing; 5) getting medical care; 6) getting service at a store or a restaurant; 7) getting credit, bank loans or a mortgage; 8) on the street or in a public setting; and 9) from the police or the courts^29^. Response options for each item included: ‘yes’, ‘no’ and ‘I do not know’. Participants who responded, ‘I do not know’ were classified as missing. All nine items were averaged for analysis, demonstrating high internal consistency (Cronbach’s α=0.903).

### Analysis

To determine whether COVID-19 influenced smoking, we used an indicator variable to distinguish the periods before and after 13 March 2020, the date when the COVID-19 pandemic was declared a national emergency in the US. First, we computed descriptive statistics for the pre- and post-pandemic samples for all sociodemographic variables, racial discrimination, depression, and cigarette use. We used two-tailed chi-squared tests and ANOVA to detect differences in demographic and outcome variables. We then conducted unadjusted and adjusted logistic regression models (sex, age, Asian ethnic group, education level, employment, and birthplace), to estimate the relationship between exposure to the COVID-19 pandemic and current smoking. Next, we examined if the relationship between exposure to COVID-19 and current smoking was modified by race/ethnicity (i.e. Chinese and South Asian). Finally, we explored whether unemployment, discrimination, and depression symptoms moderated the relationship between exposure to COVID-19 and current smoking. If the interactions were statistically significant (p<0.05), we then plotted predicted marginal probabilities of current smoking before and during the pandemic by each variable. The statistical significance was set at p<0.05 for all statistical analyses. Statistical analyses were conducted using STATA 15.

## RESULTS

**Figure 1:**
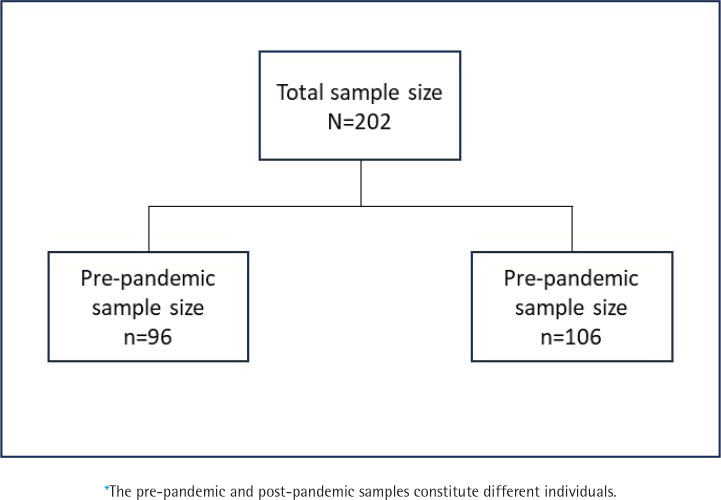
Flowchart of the study population*, Chicago Asian Health Survey 2020 (N=202)

Adults (aged ≥18 years) were eligible to participate in the survey if they resided in the Chicago land area, self-identified as South Asian or Chinese, and had smoked at least 100 cigarettes in their lifetime. From an original sample size of 209 participants, we excluded five participants with missing data for sex and two participants with missing data for education level. The final sample size for this study was 202 participants (n=96 before COVID-19; n=106 during COVID-19) (Figure 1).

This study was conducted retrospectively from data obtained for programmatic purposes. Descriptive statistics before and during the COVID-19 pandemic are shown in Table 1. Our analysis detected significant differences between the pre- and post-pandemic samples in age, Asian American ethnic group, country of birth, racial discrimination, depression symptoms, and current smoking. In particular, there were more Chinese (p<0.001) and foreign-born (p=0.008) respondents during the pandemic compared to before the pandemic. Participants were also more likely to report higher levels of racial discrimination (p<0.045) and depression symptoms (p=0.002) in the post-pandemic sample. We also found that the prevalence of current smoking (48%) was higher during the COVID-19 pandemic compared to before (28%) the pandemic (p=0.004).

**Table 1:** Study characteristics before and during COVID-19, Chicago Asian Health Survey 2020 (N=202)

*Characteristics*	*Before*	*During*
	*n (%)*	*n (%)*
**Sex**		
Male	65 (68)	75 (71)
Female	31 (32)	31 (29)
**Age** (years)		
18–29	39 (41)	22 (21)
30–65	48 (50)	69 (65)
>65	9 (9)	15 (14)
**Asian ethnic subgroups**		
Chinese	36(37)	67(63)
South Asians	60 (63)	39 (37)
**Education level**		
Lower than high school	10 (10)	22 (21)
High school	12 (12)	13 (12)
Some college	20 (21)	23 (22)
College or higher	54 (56)	48 (45)
**Employment status**		
Employed	51 (53)	65 (61)
Not employed	45 (47)	41 (39)
US born		
No	70 (73)	93 (88)
Yes	26 (27)	13 (12)
**Racial discrimination,** mean (SD)	1.07 (1.66)	2.32 (2.76)
**Depression symptoms**		
No	69 (88)	60 (68)
Yes	9 (12)	28 (32)
**Current smoking**		
No	69 (72)	55 (52)
Yes	27 (28)	51 (48)

**Figure 2:**
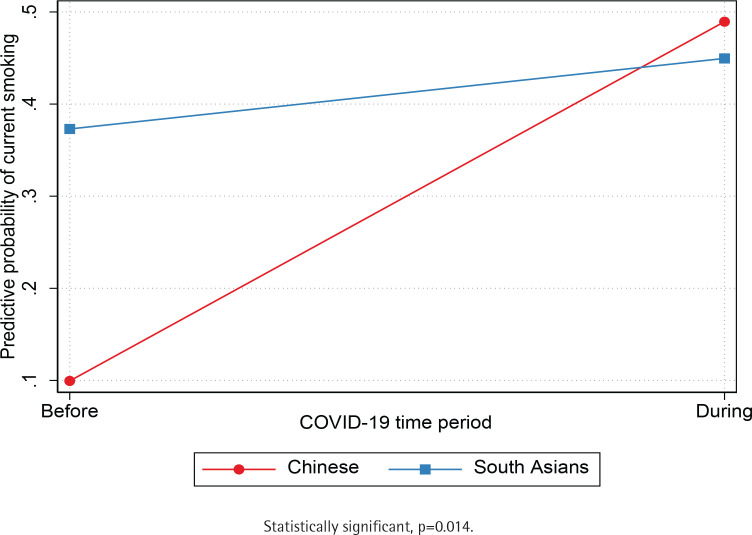
Predictive probability of current smoking before and during COVID-19 by Asian American ethnic group, Chicago Asian Health Survey 2020 (N=202)

**Figure 3:**
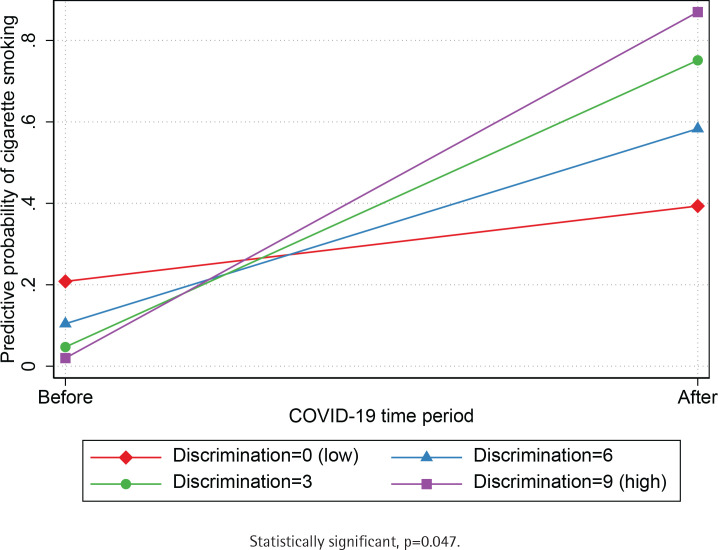
Predictive probability of discrimination before and during COVID-19 by Asian American ethnic group, Chicago Asian Health Survey 2020 (N=202)

**Figure 4:**
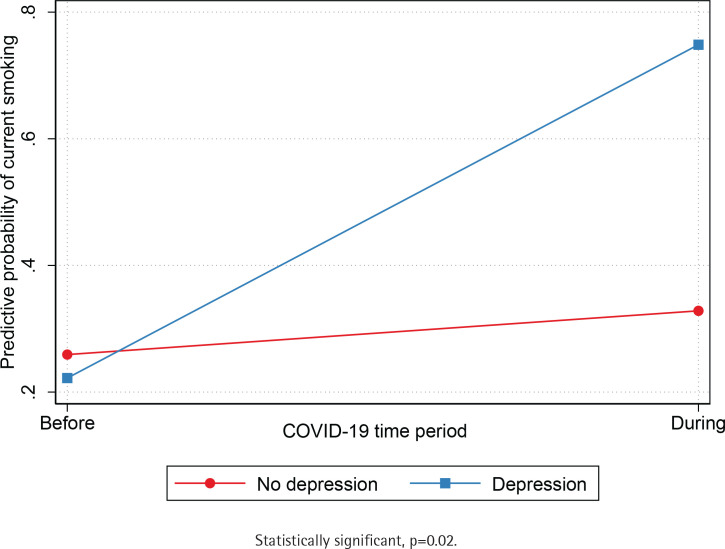
Predictive probability of depression symptoms before and during COVID-19 by Asian American ethnic group, Chicago Asian Health Survey 2020 (N=202)

Unadjusted and adjusted logistic regression models (sex, age, Asian American ethnic group, education level, employment, and birthplace) show that participants were significantly more likely to be current smokers during the COVID-19 pandemic (AOR=2.8; 95% CI: 1.37–5.71) compared to before the pandemic (Table 2). Next, we independently tested if the association between exposure to COVID-19 and current smoking was moderated by the Asian American ethnic group (Table 3). We found a statistically significant interaction between the period indicator variable and Asian American ethnic groups (p=0.014), such that the predictive probability of current smoking was lower for Chinese compared to South Asians before COVID-19 but was comparable for both groups during the pandemic (Figure 2). We also found a statistically significant interaction between the period indicator variable and racial discrimination (p=0.047) such that the predictive probability of current smoking was comparable among adults who reported low and high levels of racial discrimination before the pandemic. Still, current smoking was higher among adults who reported high levels racial discrimination during the pandemic (Figure 3). Results suggest that the predictive probability of smoking during the pandemic was progressively higher for participants who reported increased levels of racial discrimination. Finally, we found a statistically significant interaction between the COVID-19 indicator and depression symptoms (p=0.02), indicating that the predictive probability of current smoking was comparable for Asian Americans with and without depression before the outbreak of COVID-19 but higher for Asian Americans with depression during the pandemic (Figure 4).

**Table 2:** Logistic regression adjusted odds ratios of current smoking and binge drinking by sociodemographic characteristics, Chicago Asian Health Survey 2020 (N=202)^a^

*Characteristics*	*Current smoking*	
	*OR (95% CI)*	*AOR (95% CI)*
**Sex**		
Male ®		1
Female		**0.21 (0.09–0.47)**
**Age** ( years )		
18–29 ®		1
30–65		0.38 (0.14–1.01)
>65		**0.19 (0.05–0.75)**
**Asian ethnic subgroups**		
Chinese ®		1
South Asians		1.59 (0.75–3.33)
**Education level**		
Lower than high school ®		1
High school		0.97 (0.28–3.42)
Some college		1.06 (0.33–3.41)
College or higher		0.79 (0.28–2.26)
**Employment status**		
Employed ®		1
Not employed		**0.36 (0.16–0.83)**
**US born**		
No ®		1
Yes		**0.19 (0.06–0.56)**
**COVID–19 pandemic,** (%)		
Before ®	1	1
After	**2.37 (1.32–4.26)**	**1.8 (1.37–5.71)**

a The sample includes participants recruited before and during the COVID-19 pandemic. AOR: adjusted odds ratio; adjusted for sex, age, Asian ethnic group, education level, employment, and birthplace. ® Reference categories.

**Table 3:** Logistic regression adjusted odds ratios for interactions between COVID-19 time variable and sociodemographics for current smoking, Chicago Asian Health Survey 2020 (N=202)

*Current smoking interaction models*	*Discrimination*	*Employment*	*Asian ethnic subgroup*	*Depressive symptoms*
	*AOR (95%CI)*	*AOR (95%CI)*	*AOR (95%CI)*	*AOR (95%CI)*
**COVID-19 time variable** (Ref: Before)				
During	2.92 (0.97–8.77)	2.43 (1.01–5.82)	11.76 (2.93–47.11)	1.71 (0.74–3.92)
**Racial discrimination**	**0.73 (0.42–1.29)**			
COVID–19 time variable × discrimination	1.86 (1.01–3.43)			
**Employment** ( Ref: Unemployed )				
Unemployed		0.29 (0.10–0.92)		
COVID-19 time variable × employment		1.57 (0.39–6.32)		
**Asian ethnic subgroup** (Ref: Chinese)				
South Asian			6.74 (1.70–26.64)	
COVID-19 time variable × Asian ethnic subgroup			0.12 (0.02–066)	
**Depression symptoms** (Ref: No)				
Yes				0.89 (0.17–4.80)
COVID-19 time variable × depression symptoms				10.96 (1.46–82.37)
Interaction p*	**0.047**	0.526	**0.014**	**0.020**

* Each interaction was estimated from a separate logistic regression model with all main effects and a single interaction term between COVID-19 time variable and sociodemographic variable. AOR: adjusted odds ratio; models were adjusted for sociodemographic indicators.

## DISCUSSION

This is the first study that specifically examines the impact of the COVID-19 lockdown on current smoking among disaggregated data of Asian Americans (i.e. Chinese and South Asians). Consistent with previous studies^1-4^, we found that current smoking increased from 28% to 48% among Asian Americans (i.e. Chinese and South Asians) during the pandemic. However, disaggregated data by Asian American ethnic subgroups suggest that while the prevalence of current smoking was significantly lower for Chinese compared to South Asians before the pandemic, the prevalence of current smoking was similar for both groups during the pandemic. A significant increase in current smoking among Chinese (but not South Asian) during the early stages of the pandemic could reflect distress experienced by the Chinese due to the anti-Asian sentiment prevalent in the US during the pandemic^5-7^. Asian Americans have been blamed for the spread of COVID-19 as a result of divisive rhetoric reported in the media^7,30^. These results shed light on the importance of using disaggregated data to examine smoking among Asian Americans from diverse ethnic backgrounds. Moreover, collecting data from smaller geographical regions is critical to inform programmatic decisions on which populations should be targeted for smoking cessation interventions to reduce disparities. This is particularly important for South Asians and Chinese in Chicago, who smoke at significantly higher rates compared to their national counterparts (Asian Indians: 5.1%; and Chinese: 5.9%)^20^.

Results from this study suggest that Asian Americans experiencing racial discrimination during the pandemic were also more likely to be current smokers compared to their pre-pandemic counterparts. Although previous research highlights a clear relationship between racial discrimination and smoking^31-33^, this association was only evident for the post-pandemic sample in our study. This is not surprising, as the COVID-19 pandemic marked a period during which Asian Americans were exposed to increased levels of anti-Asian stigma and discrimination^5-7^. It is possible that racial discrimination during the beginning of the pandemic may have influenced smoking behavior among Asian Americans, particularly among members of disadvantaged groups. This is consistent with a recent study which found that Asian Americans who were current or former smokers were more likely to experience COVID-related discrimination (i.e. concerns that their families may be discriminated against, harassed, or treated unfairly because of the COVID-19 pandemic)^23,24^.

This study also found that the prevalence of current smoking was comparable among Asian Americans with and without depression symptoms before the pandemic, but higher for Asian Americans with depression symptoms during the pandemic. These results are consistent with a recent study conducted during the first weeks of the French lockdown that found higher smoking rates among people with depression and anxiety during the pandemic^23^. Although studies suggest that the prevalence of smoking among people with mental illness is significantly higher compared to the general population^34,35^, feelings of loneliness and isolation during COVID-19 may have an influence on smoking among people with depression. Future studies are needed to directly evaluate the influence of the pandemic on current smoking; however, it is vital that Asian American smokers receive effective smoking cessation programs that are culturally tailored to address stressors, such as discrimination and depression that may trigger cigarette use.

### Implications

Results from this study have significant implications for smoking cessation interventions among Asian American communities. For instance, considering that 66% of Asian Americans in Chicago are foreign-born^22^, it is important that smoking cessation intervention programs created for these communities are culturally and linguistically tailored to meet their needs. Future studies need to examine the attitudes and perceptions around smoking for multiple Asian American subgroups from diverse ethnic backgrounds to develop effective and culturally relevant intervention programs for these populations. Moreover, policies that address the rise in anti-Asian hate crimes and violence, need to be put in place. An online survey conducted in April 2021 found that 45% of Asian American adults reported experiencing at least one specific offensive incident associated with racial/ethnic discrimination during the COVID-19 pandemic^36^.

### Limitations

This study has several limitations that must be considered. First, the survey was a convenience sample, which may limit the generalizability of the data and result in selection bias. However, a study suggests that when engagement and recruitment of participants are done in partnership with local ethnic venues, convenience sampling can yield a representative sample of the hard-to-reach population^37^. Second, the sample size for this study was small (n=202), and we had limited power to stratify our sample and detect interactions. Third, data are cross-sectional, with two distinct cohorts of Asian Americans surveyed before and during the pandemic. Therefore, longitudinal trends in cigarette use could not be assessed, and results should be interpreted at the community level (i.e. target population level) and not at the individual level^8^. Fourth, we could not access pre- and post-pandemic changes in the number of cigarettes per day as this question was not included in the survey. Future studies should explore this association among Asian Americans, as this information is important in the development of smoking cessation programs. Fifth, residual confounding cannot be ruled out despite extensive efforts to control for known cofounders, including acculturation and country of birth. Finally, this data was collected at the beginning of the pandemic, during the national lockdown, and may not reflect other periods. Therefore, it is important to monitor smoking behavior beyond the lockdown through longitudinal studies to assess if cigarette smoking changes are sustained over time.

## CONCLUSIONS

This study contributes to our understanding of smoking behavior among Asian Americans during the initial phase of the COVID-19 pandemic, a time when this population experienced confounding stressors as a result of extreme public health measures along with exposure to anti-Asian stigma and discrimination^5-7^. Although public health measures that were put in place to prevent the spread of the virus are gradually being lifted, the impact of the COVID-19 lockdown may have lingering adverse effects on the mental health of Asian Americans, particularly those who rely on addictive substances as a coping mechanism. The present study sheds light on the importance of using disaggregated data to examine smoking and other health behaviors among Asian American populations. Results gathered from this study will be particularly helpful in informing smoking cessation programs and interventions that are culturally tailored to address the negative impact of COVID-19 and pandemic activity on smoking behavior among Asian Americans from diverse ethnic backgrounds.

## Data Availability

The data supporting this research are available from the authors on reasonable request.
